# Children with strabismus and amblyopia presented abnormal spontaneous brain activities measured through fractional amplitude of low-frequency fluctuation (fALFF)

**DOI:** 10.3389/fneur.2022.967794

**Published:** 2022-08-12

**Authors:** Xiao-Qin Hu, Yi-Dan Shi, Jun Chen, Zhipeng You, Yi-Cong Pan, Qian Ling, Hong Wei, Jie Zou, Ping Ying, Xu-Lin Liao, Ting Su, Yi-Xin Wang, Yi Shao

**Affiliations:** ^1^Department of Strabismus and Amblyopia, Affiliated Eye Hospital of Nanchang University, Nanchang, China; ^2^Department of Ophthalmology, Jiangxi Branch of National Clinical Research Center for Ocular Disease, The First Affiliated Hospital of Nanchang University, Nanchang, China; ^3^Department of Ophthalmology, The First Affiliated Hospital of Nanchang University, Jiangxi Branch of National Clinical Research Center for Ocular Disease, Nanchang, China; ^4^Department of Ophthalmology and Visual Sciences, The Chinese University of Hong Kong, Shatin, Hong Kong SAR, China; ^5^Department of Ophthalmology, Massachusetts Eye and Ear, Harvard Medical School, Boston, MA, United States; ^6^School of Optometry and Vision Sciences, College of Biomedical and Life Sciences, Cardiff University, Cardiff, United Kingdom

**Keywords:** amblyopia, fALFF, visual pathway, strabismus, children

## Abstract

**Purpose:**

Based on fMRI technology, we explored whether children with strabismus and amblyopia (SA) showed significant change in fractional amplitude of low-frequency fluctuation (fALFF) values in specific brain regions compared with healthy controls and whether this change could point to the clinical manifestations and pathogenesis of children with strabismus to a certain extent.

**Methods:**

We enrolled 23 children with SA and the same number matched healthy controls in the ophthalmology department of the First Affiliated Hospital of Nanchang University, and the whole brain was scanned by rs-fMRI. The fALFF value of each brain area was derived to examine whether there is a statistical difference between the two groups. Meanwhile, the ROC curve was made in a view to evaluate whether this difference proves useful as a diagnostic index. Finally, we analyzed whether changes in the fALFF value of some specific brain regions are related to clinical manifestations.

**Results:**

Compared with HCs, children with SA presented decreased fALFF values in the left temporal pole: the superior temporal gyrus, right middle temporal gyrus, right superior frontal gyrus, and right supplementary motor area. Meanwhile, they also showed higher fALFF values in specific brain areas, which included the left precentral gyrus, left inferior parietal, and left precuneus.

**Conclusion:**

Children with SA showed abnormal fALFF values in different brain regions. Most of these regions were allocated to the visual formation pathway, the eye movement-related pathway, or other visual-related pathways, suggesting the pathological mechanism of the patient.

## Introduction

Strabismus links to improper eye position, including esotropia and exotropia (1). The prevalence of strabismus varies in different regions or nations, but what is in common is the case that its incidence is increasing year by year (2). Refractive differences between the eyes, hyperopia, related family history, and improper behaviors during the gestation period (drinking, smoking, or drug dependence) are all clear risk factors for strabismus. Infants with congenital squint are often associated with the possibility of amblyopia, and the contemporary mainstream views are increasingly emphasizing the correction of amblyopia in children with strabismus. At the same time, strabismus constitutes one of the important reasons for the occurrence of amblyopia. The preferred practice pattern (PPP) (3) also pointed out that the correction of strabismus can promote the treatment of amblyopia. The increasing number of children with strabismus and amblyopia (SA) has grown up as a serious public health problem. Eyes are an imperative tool for directly perceiving the world. Ophthalmic abnormalities and obstacles have a profound influence on the growth process of children, and this impact is reflected in both physical development and mental development (4). Therefore, a comprehensive understanding of strabismus and amblyopia is very eminent.

Traditionally, the examinations prescribed for the diagnosis of SA were limited to optical function and acuity examinations, but a large number of studies have shown that patients with SA still have changes in the structure and function of the nervous system (5–7). However, the evaluation of this part rarely participants in clinical studies. The rapid development of the field of medical imaging in recent years suggests that we may take advantage of it to take a more intuitive and comprehensive view of SA. Among them, fMRI has become an important examination method to study the pathogenesis of SA patients at the neurological level and predict possible complications because it can evaluate the brain from many aspects such as morphology, metabolism, blood perfusion, and functional changes at the same time.

The fMRI is a noninvasive post-processing imaging technology for functional brain areas. Blood-oxygen level-dependent (BLOD) signals are obtained as being dependent upon the differences in the metabolic levels of discrete brain regions. Among them, the rs-fMRI examination is performed when the subject is awake, with closed eyes, resting, and shielded from external stimuli. The result of rs-fMRI can determine the spontaneous neural activity of the subject's brain (8).

Low-frequency oscillations (LFOs) are deemed to be caused by the spontaneous activity of brain neurons (9). When a person is in a resting state and not stimulated by the peripheral environment, there will be synchronous low-frequency vibrations in specific brain areas. This vibration can be considered by the BOLD signal, and moreover, it can be detected, recorded, and presented by rs-fMRI. On this basis, ALFF was proposed as an index of rs-fMRI. ALFF is defined as the disparity between low-frequency oscillation of BOLD signal and average fluctuation amplitude of the spontaneous brain neuron activity baseline in a specific time (10), which can be helpful to some researchers to reprocess the rs-fMRI result data for the spontaneous activity of functional brain areas (11, 12). However, in practical applications, the interference of physiological noise on the consequences of ALFF increases the uncertainty of the research results, and the improved fALFF based on ALFF can deal with this issue and remove the signal artifacts caused by non-specific brain neuronal activities.

Due to its unique advantages, fALFF has been widely used in the research of many diseases, including pure major depression disorder (13), migraine (14), post-stroke depression (15), Parkinson's disease (16), and premenstrual syndrome (17).

This study adopted rs-fMRI to detect aberrant autogenic activities in specific brain areas of patients through fALFF to explore the possible neural mechanisms and potential pathological changes in the disease. To the best of our knowledge, this is the first time that this method has been used to study SA in children.

## Materials and methods

### Subjects

The study included 23 patients (no more than18 years of age) who were diagnosed with strabismus and amblyopia and 23 healthy controls (HCs). All participants came from the Ophthalmology Department of the First Affiliated Hospital of Nanchang University.

The inclusion criteria of patients (PAT) were as follows: (1) under 18 years old; (2) being diagnosed with strabismus and amblyopia by a doctor who has obtained medical practitioner qualification strictly in accordance with the PPP diagnosis of strabismus and amblyopia (no distinction between monocular or binocular, esotropia and exotropia); (3) ophthalmoscopy showing the suppression of the macular center; and (4) lack of stereopsis.

The inclusion criteria of the healthy controls (HCs) were as follows: (1) under 18 years old; (2) non-conforming to the diagnostic criteria of strabismus and/or amblyopia; (3) could cooperate with MRI examination; (4) no history of other ophthalmic diseases; and (5) head MRI scanning showing no abnormality.

The exclusion criteria for all participants were as follows: (1) having a history of surgery, especially eye surgery; (2) having a history of eye trauma or brain trauma; (3) being born with abnormal neurological development; (4) having diseases that cannot cooperate with MRI examinations (from wearing a heart pacemaker or severe mental illness; and (5) having a history of using medications that can impact the central nervous system.

The procedure for this study was approved by the Ethics Committee of the First Affiliated Hospital of Nanchang University. All participants had known the purpose, content, and risks of this research, and written informed consent was obtained from all participants before the start of the experiment.

### MRI parameters

The MRI scan was taken using a 3.0T Siemens Trio Tim MRI Scanner (Siemens, Munich, Germany). During scanning, all participants kept their eyes closed and awake with relaxed breathing and refraining from thinking on purpose or receiving external stimulation. The supine position was maintained throughout the scan. Scanning technical parameters were as presented: repetition time (TR) = 2,000 ms, echo time (TE) = 40 ms, flip angle = 90°, acquisition matrix = 240 × 240, thickness = 4 mm, and field of view (FOV) = 240 × 240. Finally, 240 functional images were generated. The entire scanning time went on for 8 min. The scanning range covered the entire brain.

### fMRI data analysis

The MRIcro software was adopted to classify, identify, and delete incomplete data from the MRI scan for the integrity and validity of the data. The utilization of Statistical Parametric Mapping software8 (SPM8) was used for data preprocessing, which includes (1) abandon the first 15 functional images, convert the remaining data to the NIFTI format, and perform time layer correction and head movement correction (retaining the data with head movement ≤1.5 mm and head rotation ≤2°); (2) taking into account the difference in brain volume of different subjects, standardize and resemble the fMRI images using echo plane imaging templates [using the Montreal Institute of Neurology (MNI) spatial standard; voxel = 3 mm × 3 mm × 3 mm]; (3) eliminate the linear trend of the time series and perform low-frequency filtering (0.01–0.08 Hz) on it to reduce low-frequency drift and high-frequency noise; and (4) use full width and half height (FWHM: 6 × 6 × 6 mm) to smooth the image.

The covariance referenced in the regression analysis contains the 6 parameters of head movement, the average frame displacement [FD], the overall brain signal, and the average signal of white matter and cerebrospinal fluid.

### Calculation of fALFF values

The value of fALFF is equal to the ratio of the power spectrum in a specific low-frequency range of a certain brain area to the power spectrum of the entire frequency range, which can reduce the interference caused by the normal physiological activities of other brain areas to a certain extent. The REST software was selected for the calculation, conversion of time series data and a restricted frequency range, and calculation of the power spectrum. The specific low-frequency range is set to 0.01–0.08 Hz, and the entire frequency range is set to 0–0.25 Hz.

### Correlation analysis

Correlation analysis was carried out to evaluate the relationship between typical autonomous activities and clinical performance. Indexes we adopted included the minimum resolution angle logarithmic vision (LogMAR), which is based on the value of best-corrected visual acuity (BCVA); and the hospital anxiety and depression scale (HADS), which could quantify and evaluate the level of anxiety. With the application of the REST software, we defined brain regions in PAT with different fALFF values as regions of interest (ROI) and analyzed the correlation between mean fALFF values of ROI and one of the indexes through linear correlation analysis (α = 0.05, *p* < α is statistically significant).

### Statistical analysis

The statistical analysis involved in the experiment was carried out using SPSS 20.0 (SPSS, IBM Corp, USA). General information and clinical characteristics of the subjects were statistically tested by independent sample *t*-test and chi-square test (α = 0.05, *p* < α suggests that the difference is statistically significant). The difference of fALFF between PAT and HCs was verified by two independent sample *t*-tests to determine if it is statistically significant, and the value of this difference as a diagnostic indicator was analyzed by the ROC curve. The correlation analysis between the fALFF value and clinical manifestations of specific brain areas in the PAT group was performed by Pearson's correlation analysis (α = 0.05, *p* < α is statistically significant).

## Results

### Demographics and visual measurements

There was no significant difference in gender (*p* > 0.99), age (*p* = 0.322), and BCVA (*p* = 0.276 for domain eyes and *P* = 0.295 for the fellow eye). More details are given in Table 1.

**Table 1:** The conditions of participants included in the study.

**Condition**	**SA**	**HCs**	***t*-value**	*P*-value*
Male/female Age (years) Weight (kg) Handedness Best-corrected VA-DE Best-corrected VA-FE Duration of SA (years) Esotropia/exotropia	15/8 10.46 ± 1.29 28.53 ± 3.64 23R 1.15 ± 0.15 1.10 ± 0.15 10.46 ± 1.29 13/10	15/8 11.61 ± 1.32 29.64 ± 3.54 23R 1.10 ± 0.10 1.15 ± 0.10 N/A N/A	N/A −1.056 −0.784 N/A 1.875 1.864 N/A N/A	>0.99 0.322 0.595 >0.99 0.276 0.295 N/A N/A
Angle of strabismus (PD)	37.39 ± 9.24	N/A	N/A	N/A

Independent t-tests comparing two groups (p < 0.05 represented statistically significant differences).

DE, dominant eye; FE, fellow eye; HCs, healthy controls; N/A, not applicable; PD, prism diopter; SA, strabismus and amblyopia; VA, visual acuity.

### fALFF differences

**Figure 1:**
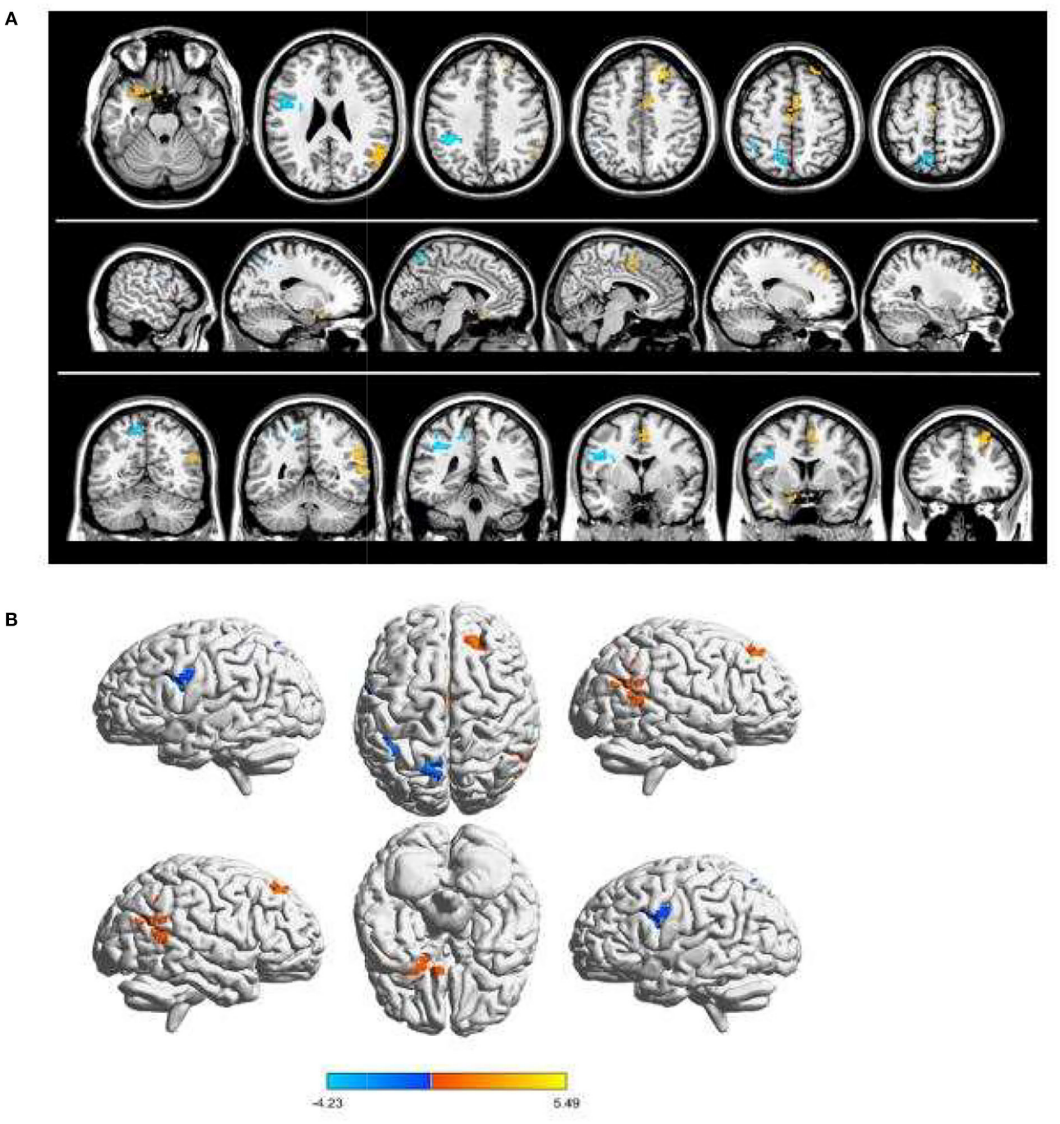
Spontaneous brain activity in children and HCs. **(A)** The brain regions presented sensible differences in fAlFF values between SA and HCs. **(B)** Children with SA would be observed some abnormal brain activities in specific regions. Compared with HCs, red regions trend to mean higher fALFF values. Regions marked by blue presented delegated regions presenting decreased fALFF values. fALFF, fractional amplitude of low-frequency fluctuation; HCs, healthy controls; SA, strabismus and amblyopia.

**Figure 2:**
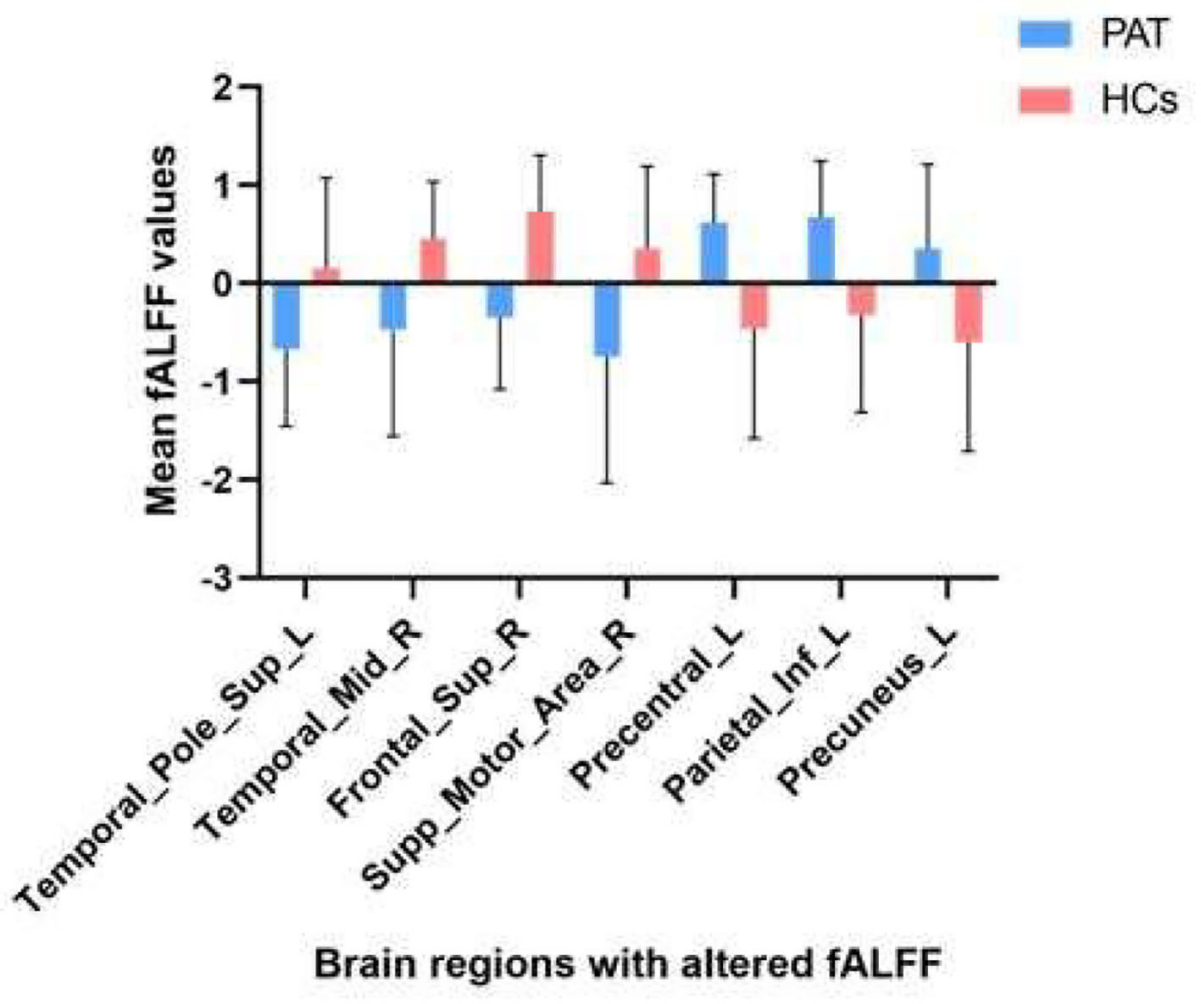
The mean fALFF values between children with SA and HCs.

Children with SA had decreased fALFF values in Temporal-Pole-Sup-L, Temporal-Mid-R, Frontal-Sup-R, and Supp-Motor-Area-R. Moreover, they also presented higher fALFF values in specific brain areas, which included Precentral-L, Precental gyrus, Parietal_Inf-L, and Precuneus-L. More detailed information is shown in Figure 1 and Table 2. In the meantime, Figure 2 depicts the mean of changed spontaneous brain activity between PAT and HCs.

**Table 2:** Brain regions with significant differences in fALFF between PAT and HC groups.

**Brain areas**	**MNI coordinates**			**fALFF**			**ROI sequence**
	**X**	**Y**	**Z**	**BA**	**Peak voxels**	***t*-value**	
**HCs>PAT**							
Temporal_Pole_Sup_L	−15	9	−24	47	77	3.28	Cluster 1
Temporal_Mid_R	60	−51	9	22	89	3.54	Cluster 3
Frontal_Sup_R	21	33	45	8	65	5.49	Cluster 5
Supp_Motor_Area_R	3	0	54	6	59	3.37	Cluster 6
**HCs < PAT**							
Precentral_L	−30	−3	21	6	99	−4.23	Cluster 2
Parietal_Inf_L	−39	−39	39	40	64	−4.16	Cluster 4
Precuneus_L	−9	−60	60	7	66	−3.2	Cluster 7

α = 0.05 for multiple comparisons through Gaussian random field theory (z > 2.3, p < 0.01, cluster size > 40 voxels, alphasim corrected).

PAT, patients; HCs, healthy controls; MNI, Montreal Neurological Institute; BA, Brodmann area; ROI, region of interest; L, left; R, right; Temporal-Pole-Sup, Temporal pole: superior temporal gyrus; Temporal-Mid, middle temporal gyrus; Frontal-Sup, superior frontal gyrus; Supp-Motor-Area, supplementary motor area; Precentral, precentral gyrus; Parietal_Inf, inferior parietal, but supramarginal and angular gyri.

### ROC analysis

**Figure 3:**
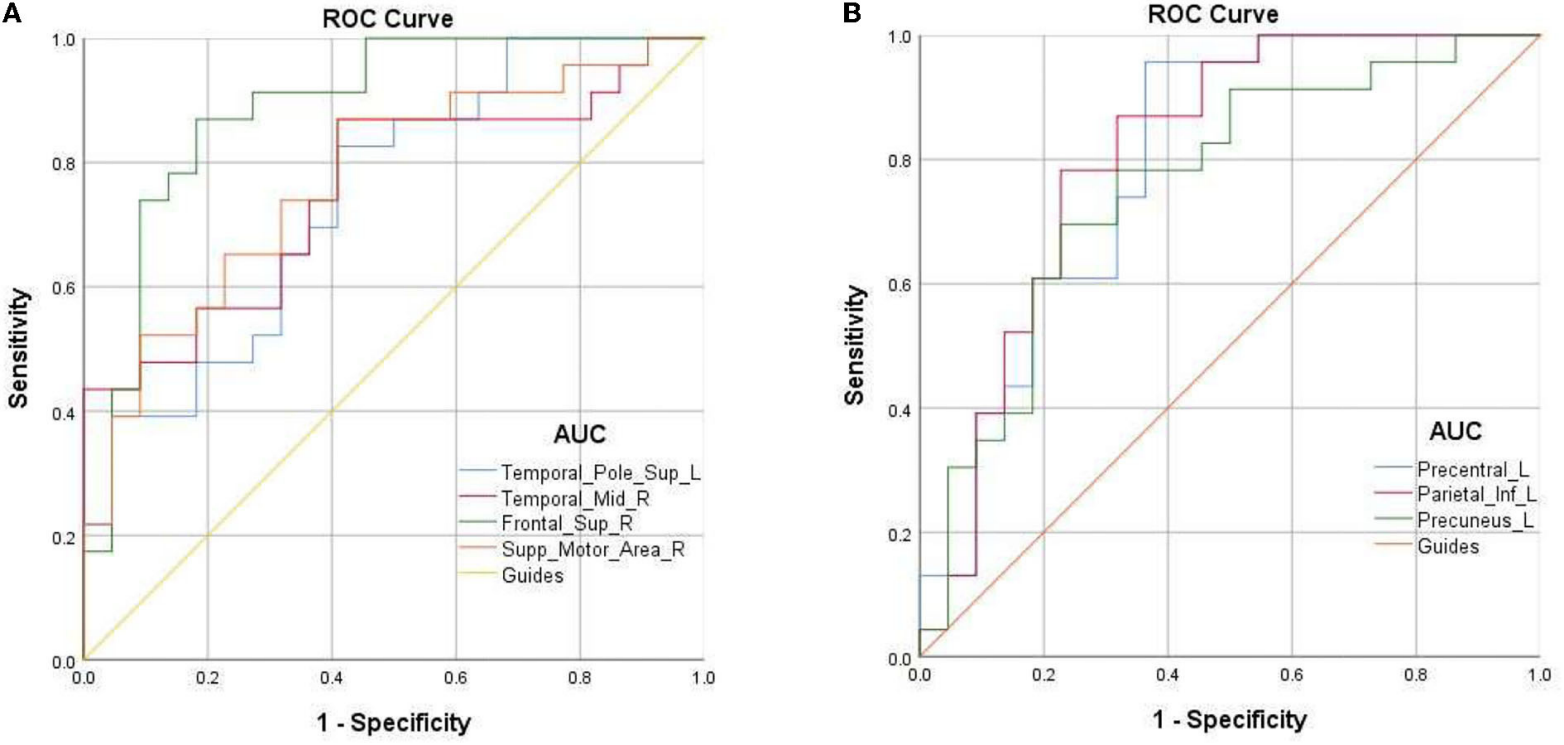
ROC curve analysis for the mean fALFF values of altered brain regions. **(A)** The area under ROC curve was 0.745 for Temporal-Pole-Sup-L (*p* = 0.005, 95%CI:0.603–0.887); 0.755 for Temporal-Mid-R (*p* = 0.003, 95%CI:0.621–0.898); 0.887 for Frontal-Sup-R (*p* < 0.001, 95%CI: 0.787–0.988); 0.773 for Supp-Motor-Area-R (*p* = 0.002, 95%CI: 0.635–0.910). **(B)** The area under ROC curve was 0.69 for Precentral-L (*p* = 0.001, 95%CI:0.659–0.930); 0.68 for Parietal-L (*p* < 0.001, 95%CI: 0.675–0.941); 0.73 for Precuneus-L (*p* = 0.003, 95%CI: 0.616–0.902).

Due to the difference in fALFF values in some brain regions between PAT and SA, we wonder whether this discrepancy could be considered as a diagnostic criterion for SA, and thus we chose the receiver operating characteristic (ROC) curve as a common method to explore the diagnostic value of this difference. We focused on the area under the curve (AUC) of the ROC curve, because this indicator can simultaneously take sensitivity and specificity into account. We divided the accuracy into low (AUC0.5–0.7) and high (AUC0.7–0.9) levels to evaluate its diagnostic value more accurately. The AUC value is 0.745 for Temporal-Pole-Sup-L (*p* = 0.005); 0.755 for Temporal-Mid-R (*p* = 0.003); 0.887 for Frontal-Sup-R (*p* < 0.001); 0.773 for Supp-Motor-Area-R (*p* = 0.002); 0.69 for Precentral-L (*p* = 0.001); 0.68 for Parietal-L (*p* < 0.001); and 0.73 for Precuneus-L (*P* = 0.003) (Figure 3).

### Correlation analysis

**Figure 4:**
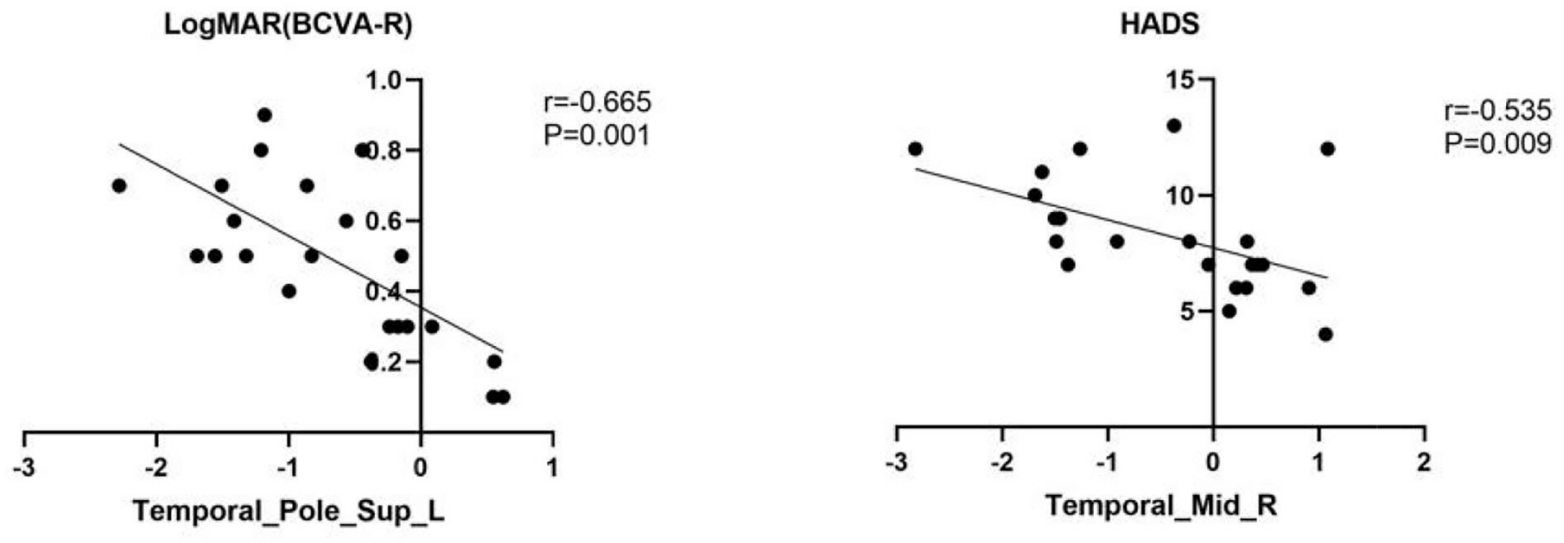
The result of correlation analysis. The values of LogMAR(BCVA-R) of PAT presented a significant correlation with the fALFF values of Temporal-Pole-Sup-L. The scores of HADS showed a negative correlation with fALFF values of temporal_mid_R (*r* = −0.535, *p* = 0.009).

Mean fALFF values of temporal-pole-sup-L showed a negative correlation with log MAR (*r* = −0.665, *p* = 0.001). Meanwhile, a familiar correlation was found between temporal-mid-R and HADS (*r* = −0.535, *p* = 0.009) (Figure 4).

**Figure 5:**
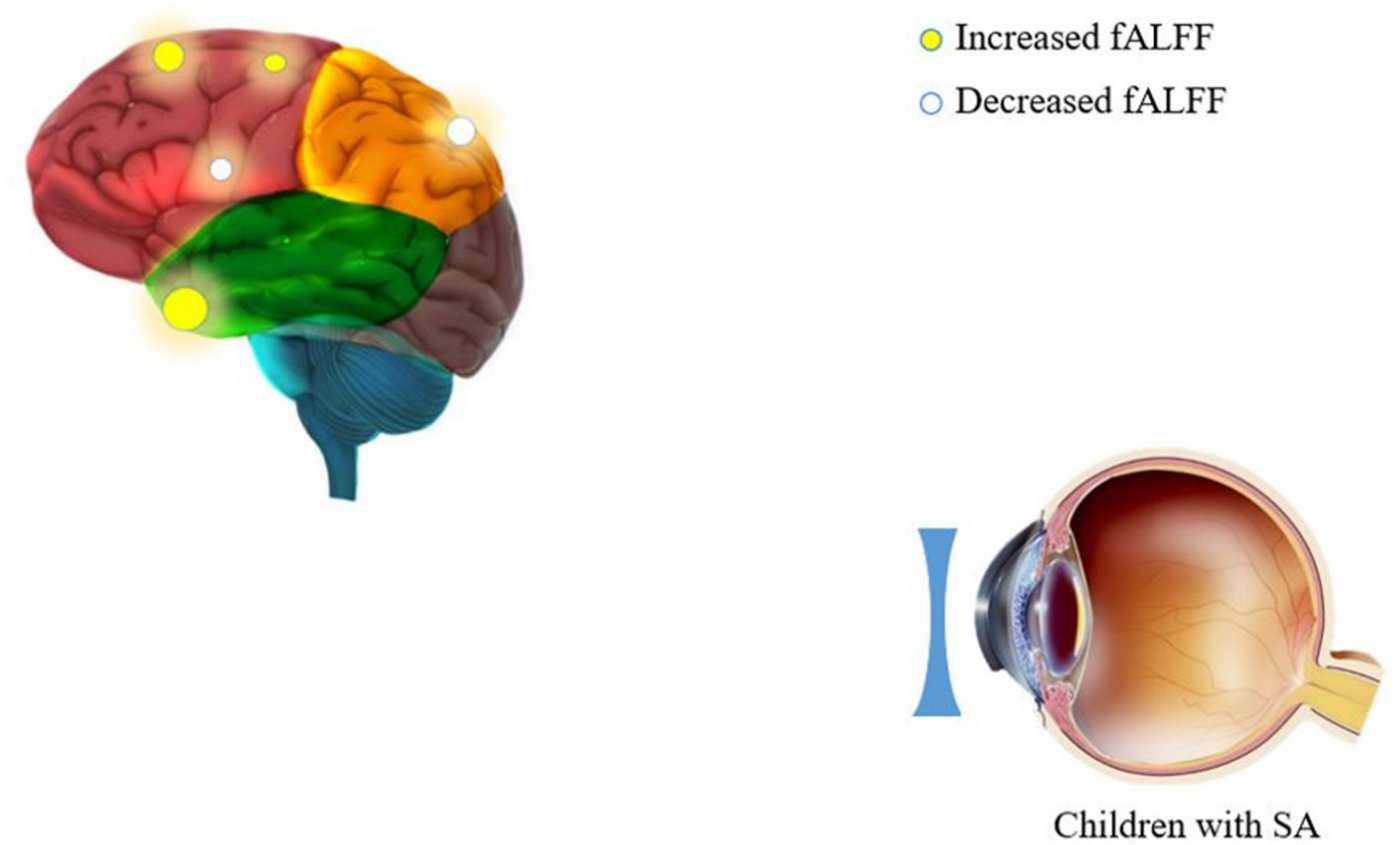
Increase and decrease areas of flaff of children with SA.

## Discussion

### Analysis of the increased fALFF in children with SA

The superior temporal gyrus is associated with language comprehension (18), visual search (19), and other functions. The bilateral superior temporal gyrus and middle temporal gyrus are also known as the V5/MT area (visual area 5/middle temporal gyrus), and the functional connections in the hippocampus play an important role in visual memory (20). The V5/MT area is also the core area of the global motion perception (GMP) (21), that is, in a specific visual scene, the motion trajectory of a single element is integrated to form a comprehensive three-dimensional stimulus. In many diseases, there are certain pathological changes in the superior temporal gyrus, including schizophrenia (22), Alzheimer's disease (23), adult common exotropia (24) and unilateral acute open eye injury (25). Wang et al. (26) found that the thickness of the intra cellular in the V5/MT area of patients with high intraocular pressure glaucoma was reduced, which may be related to the decrease in the high intraocular pressure and visual stimulation caused by the disease (27, 28). The study of Cai et al. (29) showed that the stimulation of the V5/MT area may cause the subjects to discriminate the overall direction of movement. Like this result, the increase in the fALFF value in this experiment indicates that the V5/MT area of SA patients is overactive, which may be related to the compensatory overestimation of this area caused by the obstacle of SA patients' judgment of spatial location.

The frontal lobe is one of the main functional areas of the cerebral hemisphere, and there are aberrant immunological changes in the superior frontal gyrus in many ophthalmological diseases. Huang et al. (30) showed that the ALFF value of the superior frontal gyrus of patients with primary angle-closure glaucoma (POAG) decreased, and the increase in the ALFF value of the superior frontal gyrus was also discovered in patients with corneal ulcer (31). Adults with SA also showed that the ALFF on the right forehead is worth increasing (6). This phenomenon may be due to the frontal eye field (FEF) (23, 32) formed by the autophagy of the frontal gyrus, which is related to saccade movement, visual perception (32), and pain (33). In this study, children with strabismus and amblyopia also showed an increase in the fALFF value of the right superior frontal gyrus, suggesting that compared with HCs, the spontaneous activities of the right superior frontal gyrus of PAT were more active and caused eye movement disorders and vision problems in patients with SA. The ability to receive and integrate stimuli decreases as the outcome of compensatory hyperfunction of the superior frontal gyrus.

The supplementary motor area (SMA) includes a part of the side of Brodmand 6 and 8. The anterior extremity is the supplementary eye-field (SEF) and is next to the supplementary sensory area. Stimulating SEF under laboratory conditions could cause eye movement and combined eye movement (34). It shows that spontaneous brain activities of SEF could be detected before the movement of the unilateral eyeball (35), and there will be SMA activation after showing the intention to change the existing combined eye movement state (36). Discrete lesions of SEF and SMA can cause abnormal eye movements in patients (37). In addition, activation of SMA can also be observed in sequence learning (38). Studies believe that this activation is explained by the visual cues and responses required during the learning process (39). At present, it is believed that the post-spinal inhibition of the supplemental exercise area involved in exercise is closely connected with diseases such as Parkinson's disease (40). The SMA area, especially the SEF area, is closely connected with the movement of the eyeball. In this study, we found that there is a decrease in fALFF value in the SMA area in children with SA, which may indicate that in the early stages of the course of strabismus in children, there is a functional compensation in this brain area due to abnormal eye movements, thus showing unusually active.

### Analysis of the decreased fALFF in PAT

The precentral gyrus is part of the primary motor cortex (41), which receives proprioception and regulates autonomous movement. Studies have found that in many ophthalmological diseases, changes in the structure and function of the precentral can be observed. Huang et al. (30) found that the ALFF value of precentral in PACG patients decreased, and analogously, Chan et al. (42) showed that the gray matter volume (GMV) of the right precentral gyrus was increased in patients with strabismus. The study by Lin et al. (43) observed more active spontaneous brain activities in the precentral gyrus in anisometropia patients. Those conclusions are in agreement with the results of our study, suggesting that children with SA have spontaneous eye movement disorders.

The parietal lobe is related to higher cognitive functions and thinking processing (44), while the inferior parietal lobules are thought to be related to the oculomotor nerve, the forming and maintaining of attention, hand-eye coordination recalibration (45), and language learning in real life (46). Meanwhile, it was also reported to be greatly helpful to choose information, which is related to visual space (47). In this study, the fALFF value of the inferior parietal reduced, which may be related to the abnormal ocular function increasing the obstacles of language learning in children, leading to the lack of reading and spelling ability.

The default mode network (DMN) refers to a functional network composed of brain regions that are spatially separated but show a high degree of temporal correlation at rest. DMN involves numerous brain regions, including the subcortical region of the parietal lobe, middle frontal gyrus, frontal gyrus, and precuneus (48).

The precuneus plays an essential role in DMN and participates in the formation of optical network pathways. It also plays an irreplaceable role in the visual spatial imaging (49), self-processing (50), episodic memory extraction (51), spatial position coding (52), etc. We reviewed the studies of other researchers and found that many eye diseases have been observed to change the structure or connection function and spontaneous activity of the precuneus. In patients with binocular blindness, the volume of local gray matter in the precuneus boils down (17). Further studies have shown that in normal-tension glaucoma (53), diabetic retinopathy (54), primary angle-closure glaucoma (30), and other diseases, the precuneus shows spontaneous reduction of brain activity, which is consistent with our research. The conclusions are the same, and the results of the study are also consistent with the clinical manifestations of SA children with eye movement disorders and abnormal visual spatial imaging. However, Tan et al. (55) found that with withdrawnness lobe injury (OGI), the ALFF value associated with recuneus was increased, which was negatively related to the duration of OGI. This result strongly suggested that in the primary stage of eye injury, the damage will be compensated by more vigorous spontaneous brain activities of the precuneus. However, as the course of the disease continues, this compensatory effect may be lowered. This hypothesis can also explain the consequences of some parallel studies that are contrary to the results of our study (30).

In addition, due to the superficial anatomical position of the eyes, it is not difficult to notice in daily life and interpersonal communication. Therefore, some scholars believe that strabismus is actually a cosmetic disease (4), and childhood is an important period of character formation and interpersonal communication. Therefore, congenital SA may influence the physical and mental health of children. Studies have proved that SA may cause patients to feel destructive emotions such as low self-esteem and anxiety (56). In our study, the HADS was used for evaluating the anxiety of children, and it was found that the HADS score of SA patients was negatively correlated with the spontaneous brain activity of the middle temporal gyrus. This anxiety may be secondary to the decreased activity of the middle temporal gyrus area in SA disease, or it may be attributable to the disease making children become unconfident and anxious in everyday life and social activities (Figure 3 and Table 3).

**Table 3:** The function of brain regions with altered fALFF values and its clinical significance.

**Brain region**	**Experiment result**	**Function**	**Anticipated results**
Temporal-Pole-Sup-L	HC>PAT	Auditognosis; Language; emotion processing	Depression; anxiety; visual impairment
Temporal-Mid-R	HC>PAT	Forming DMN; recognition and processing of color and shape	Depression; anxiety
Frontal-Sup-R	HC>PAT	Memory; processing of cognitive information	Damaged spatial cognitive ability and eye-hand coordination
Supp-Motor-Area-R	HC>PAT	Action inhibition; modulating interhemispheric interactions	Epilepsy; depression; motor neglect
Precentral-L	HC < PAT	Somatic movement controlling	Damaged visual function
Parietal-Inf-L	HC < PAT	Part of DMN; Advanced cognitive function	Depression; anxiety
Precuneus-L	HC < PAT	Visuospatial imagery; attention; episodic memory; Functional core of DMN; consciousness	Pain felling; dysfunction of spatial orientation

PAT, patient; HC, healthy controls; DMN, default-mode network.

It should be noted in particular that there are still some limitations in this study, including (1) samples included in the study are not adequate; (2) the subjects were younger, and there may be a low degree of coordination in the process of fMRI examination; and (3) mixed bias is unavoidable.

## Conclusion

In this study, we found that children with SA presented abnormal spontaneous brain activities in the visual pathway or visual-related brain regions. These abnormal spontaneous activities may impact on the patient's clinical manifestations or be attributed to compensatory eye movement dysfunction. Besides, correlation analysis also showed that SA in childhood may cause undesirable emotions in patients.

## Data availability statement

The raw data supporting the conclusions of this article will be made available by the authors, without undue reservation.

## Ethics statement

The studies involving human participants were reviewed and approved by the committee of the medical ethics of the First Affiliated Hospital of Nanchang University. The patients/participants provided their written informed consent to participate in this study.

## Author contributions

X-QH, Y-DS, and JC were major contributors, conceived and designed the experiments, analyzed the data, and wrote and revised the manuscript. ZY, Y-CP, QL, HW, and JZ recruited the patients and healthy controls for the study and performed the MRI experiments. PY, X-LL, TS, and Y-XW collected and treated the data. YS designed the study and obtained financial support. All the authors read and approved the final manuscript.

## Funding

National Natural Science Foundation (No: 82160195); Central Government Guides Local Science and Technology Development Foundation (No: 20211ZDG02003); Key Research Foundation of Jiangxi Province (Nos: 20181BBG70004 and 20203BBG73059).

## Conflict of interest

The authors declare that the research was conducted in the absence of any commercial or financial relationships that could be construed as a potential conflict of interest.

## Publisher's note

All claims expressed in this article are solely those of the authors and do not necessarily represent those of their affiliated organizations, or those of the publisher, the editors and the reviewers. Any product that may be evaluated in this article, or claim that may be made by its manufacturer, is not guaranteed or endorsed by the publisher.

## References

[1] Sloper J. The other side of amblyopia. JAAPOS. (2016) 20:1. 10.1016/j.jaapos.2015.09.013 26917086

[2] Roider L Ungerer G Shock L Aldridge K Al–Samarraie M Tanaka T. Increased incidence of ophthalmologic findings in children with concurrent isolated non-syndromic metopic suture abnormalities and deformational cranial vault asymmetry. Cleft Palate Craniofac J. (2021) 58:497–504. 10.1177/1055665620954739 32929979

[3] Wallace DK Christiansen SP Sprunger DT Melia M Lee KA Morse CL. Esotropia and exotropia preferred practice pattern(R). Ophthalmology. (2018) 125:P143–83. 10.1016/j.ophtha.2017.10.007 29108746

[4] Menon V Saha J Tandon R Mehta M Khokhar S. Study of the psychosocial aspects of strabismus. J Pediatr Ophthalmol Strabismus. (2002) 39:203–8. 10.3928/0191-3913-20020701-07 12148552

[5] Shao Y Li QH Li B Lin Q Su T Shi WQ. Altered brain activity in patients with strabismus and amblyopia detected by analysis of regional homogeneity: a resting state functional magnetic resonance imaging study. Mol Med Rep. (2019) 19:4832–40. 10.3892/mmr.2019.10147 31059016 PMC6522834

[6] Min YL Su T Shu YQ Liu WF Chen LL Shi WQ. Altered spontaneous brain activity patterns in strabismus with amblyopia patients using amplitude of low–frequency fluctuation: a resting–state fMRI study. Neuropsychiatr Dis Treat. (2018) 14:2351–2359. 10.2147/NDT.S171462 30275692 PMC6157537

[7] Joly O Franko E. Neuroimaging of amblyopia and binocular vision: a review. Front Integr Neurosci. (2014) 8:62. 10.3389/fnint.2014.00062 25147511 PMC4123726

[8] Bullmore E Sporns O. Complex brain networks: graph theoretical analysis of structural and functional systems. Nat Rev Neurosci. (2009) 10:186–198. 10.1038/nrn2575 19190637

[9] Maknojia S Churchill NW Schweizer TA Graham SJ. Resting state fMRI: going through the motions. Front Neurosci. (2019) 13:825. 10.3389/fnins.2019.00825 31456656 PMC6700228

[10] Zang YF He Y Zhu CZ Cao QJ Sui MQ Liang M. Altered baseline brain activity in children with ADHD revealed by resting–state functional MRI. Brain Dev. (2007) 29:83–91. 10.1016/j.braindev.2006.07.002 16919409

[11] Shmuel A Leopold DA. Neuronal correlates of spontaneous fluctuations in fMRI signals in monkey visual cortex: implications for functional connectivity at rest. Hum Brain Mapp. (2008) 29:751–761. 10.1002/hbm.20580 18465799 PMC6870786

[12] Jiang GH Qiu YW Zhang XL Han LJ Lv XF Li LM. Amplitude low–frequency oscillation abnormalities in the heroin users: a resting state fMRI study. Neuroimage. (2011) 57:149–154. 10.1016/j.neuroimage.2011.04.004 21515385

[13] Wang ZL Zou L Lu ZW Xie XQ Jia ZZ Pan CJ. Abnormal spontaneous brain activity in type 2 diabetic retinopathy revealed by amplitude of low–frequency fluctuations: a resting–state fMRI study. Clin Radiol. (2017) 72:340–341. 10.1016/j.crad.2016.11.012 28041652

[14] Bruner E Preuss TM Chen X Rilling JK. Evidence for expansion of the precuneus in human evolution. Brain Struct Funct. (2017) 222:1053–1060. 10.1007/s00429-015-1172-y 26725108 PMC4930733

[15] Ye Q Zou F Lau H Hu Y Kwok SC. Causal evidence for mnemonic metacognition in human precuneus. J Neurosci. (2018) 38:6379–6387. 10.1523/JNEUROSCI.0660-18.2018 29921714 PMC6041789

[16] Cavanna AE. The precuneus and consciousness. CNS Spectr. (2007) 12:545–552. 10.1017/s1092852900021295 17603406

[17] Wan CY Wood AG Chen J Wilson SJ Reutens DC. The influence of preterm birth on structural alterations of the vision–deprived brain. Cortex. (2013) 49:1100–9. 10.1016/j.cortex.2012.03.013 22591801

[18] Bigler ED Mortensen S Neeley ES Ozonoff S Krasny L Johnson M. Superior temporal gyrus, language function, and autism. Dev Neuropsychol. (2007) 31:217–238. 10.1080/87565640701190841 17488217

[19] Gharabaghi A Fruhmann BM Tatagiba M Karnath HO. The role of the right superior temporal gyrus in visual search–insights from intraoperative electrical stimulation. Neuropsychologia. (2006) 44:2578–81. 10.1016/j.neuropsychologia.2006.04.006 16750545

[20] Murray EA Mishkin M. Severe tactual as well as visual memory deficits follow combined removal of the amygdala and hippocampus in monkeys. J Neurosci. (1984) 4:2565–80. 10.1523/JNEUROSCI.04-10-02565.1984 6491723 PMC6564694

[21] Chen N Cai P Zhou T Thompson B Fang F. Perceptual learning modifies the functional specializations of visual cortical areas. Proc Natl Acad Sci U S A. (2016) 113:5724–9. 10.1073/pnas.1524160113 27051066 PMC4878474

[22] Kasai K Shenton ME Salisbury DF Hirayasu Y Onitsuka T Spencer MH. Progressive decrease of left Heschl gyrus and planum temporale gray matter volume in first–episode schizophrenia: a longitudinal magnetic resonance imaging study. Arch Gen Psychiatry. (2003) 60:766–75. 10.1001/archpsyc.60.8.766 12912760 PMC2901861

[23] Watson CT Roussos P Garg P Ho DJ Azam N Katsel PL. Genome–wide DNA methylation profiling in the superior temporal gyrus reveals epigenetic signatures associated with Alzheimer's disease. Genome Med. (2016) 8:5. 10.1186/s13073-015-0258-8 26803900 PMC4719699

[24] Tan G Dan ZR Zhang Y Huang X Zhong YL Ye LH. Altered brain network centrality in patients with adult comitant exotropia strabismus: a resting–state fMRI study. J Int Med Res. (2018) 46:392–402. 10.1177/0300060517715340 28679330 PMC6011327

[25] Huang X Li HJ Ye L Zhang Y Wei R Zhong YL. Altered regional homogeneity in patients with unilateral acute open–globe injury:a resting–state functional MRI study. Neuropsychiatr Dis Treat. (2016) 12:1901–6. 10.2147/NDT.S110541 27536111 PMC4975161

[26] Wang Y Wang X Zhou J Qiu J Yan T Xie Y. Brain morphological alterations of cerebral cortex and subcortical nuclei in high–tension glaucoma brain and its associations with intraocular pressure. Neuroradiology. (2020) 62:495–502. 10.1007/s00234-019-02347-1 31872278

[27] Li C Cai P Shi L Lin Y Zhang J Liu S. Voxel–based morphometry of the visual–related cortex in primary open angle glaucoma. Curr Eye Res. (2012) 37:794–802. 10.3109/02713683.2012.683506 22631870

[28] Yu L Xie B Yin X Liang M Evans AC Wang J. Reduced cortical thickness in primary open–angle glaucoma and its relationship to the retinal nerve fiber layer thickness. PLoS ONE. (2013) 8:e73208. 10.1371/journal.pone.0073208 24019910 PMC3760921

[29] Cai P Chen N Zhou T Thompson B Fang F. Global versus local:double dissociation between MT+ and V3A in motion processing revealed using continuous theta burst transcranial magnetic stimulation. Exp Brain Res. (2014) 232:4035–41. 10.1007/s00221-014-4084-9 25200175

[30] Huang X Zhong YL Zeng XJ Zhou F Liu XH Hu PH. Disturbed spontaneous brain activity pattern in patients with primary angle–closure glaucoma using amplitude of low–frequency fluctuation: a fMRI study. Neuropsychiatr Dis Treat. (2015) 11:1877–83. 10.2147/NDT.S87596 26251603 PMC4524585

[31] Shi WQ Wu W Ye L Jiang N Liu WF Shu YQ. Altered spontaneous brain activity patterns in patients with corneal ulcer using amplitude of low–frequency fluctuation: an fMRI study. Exp Ther Med. (2019) 18:125–132. 10.3892/etm.2019.7550 31258645 PMC6566102

[32] Paus T. Location and function of the human frontal eye–field: a selective review. Neuropsychologia. (1996) 34:475–83. 10.1016/0028-3932(95)00134-4 8736560

[33] Obermann M Rodriguez–Raecke R Naegel S Holle D Mueller D Yoon MS. Gray matter volume reduction reflects chronic pain in trigeminal neuralgia. Neuroimage. (2013) 74:352–358. 10.1016/j.neuroimage.2013.02.029 23485849

[34] Martinez–Trujillo JC Medendorp WP Wang H Crawford JD. Frames of reference for eye–head gaze commands in primate supplementary eye fields. Neuron. (2004) 44:1057–66. 10.1016/j.neuron.2004.12.004 15603747

[35] Schlag J Schlag–Rey M. Evidence for a supplementary eye field. J Neurophysiol. (1987) 57:179–200. 10.1152/jn.1987.57.1.179 3559671

[36] Li CS Huang C Constable RT Sinha R. Imaging response inhibition in a stop–signal task: neural correlates independent of signal monitoring and post–response processing. J Neurosci. (2006) 26:186–192. 10.1523/JNEUROSCI.3741-05.2006 16399686 PMC6674298

[37] Nachev P Kennard C Husain M. Functional role of the supplementary and pre–supplementary motor areas. Nat Rev Neurosci. (2008) 9:856–69. 10.1038/nrn2478 18843271

[38] Hikosaka O Sakai K Miyauchi S Takino R Sasaki Y Putz B. Activation of human pre-supplementary motor area in learning of sequential procedures: a functional MRI study. J Neurophysiol. (1996) 76:617–621. 10.1152/jn.1996.76.1.617 8836248

[39] Sakai K Hikosaka O Miyauchi S Sasaki Y Fujimaki N Putz B. Presupplementary motor area activation during sequence learning reflects visuo–motor association. J Neurosci. (1999) 19:C1. 10.1523/JNEUROSCI.19-10-j0002.1999 PMC6782738 10234047

[40] Botzel K Schulze S. Self–initiated versus externally triggered movements. I. An investigation using measurement of regional cerebral blood flow with PET and movement–related potentials in normal and Parkinson's disease subjects. Brain. (1996) 119 (Pt 3):1045–8. 10.1093/brain/119.3.1045 8673482

[41] Binkofski F Fink GR Geyer S Buccino G Gruber O Shah NJ. Neural activity in human primary motor cortex areas 4a and 4p is modulated differentially by attention to action. J Neurophysiol. (2002) 88:514–9. 10.1152/jn.2002.88.1.514 12091573

[42] Chan ST Tang KW Lam KC Chan LK Mendola JD Kwong KK. Neuroanatomy of adult strabismus: a voxel–based morphometric analysis of magnetic resonance structural scans. Neuroimage. (2004) 22:986–994. 10.1016/j.neuroimage.2004.02.021 15193630

[43] Lin X Ding K Liu Y Yan X Song S Jiang T. Altered spontaneous activity in an isometropic amblyopia subjects: revealed by resting–state FMRI. PLoS ONE. (2012) 7:e43373. 10.1371/journal.pone.0043373 22937041 PMC3427333

[44] Catani M Robertsson N Beyh A Huynh V de Santiago RF Howells H. Short parietal lobe connections of the human and monkey brain. Cortex. (2017) 97:339–357. 10.1016/j.cortex.2017.10.022 29157936

[45] Clower DM West RA Lynch JC Strick PL. The inferior parietal lobule is the target of output from the superior colliculus, hippocampus, and cerebellum. J Neurosci. (2001) 21:6283–91. 10.1523/JNEUROSCI.21-16-06283.2001 11487651 PMC6763148

[46] Sliwinska MW James A Devlin JT. Inferior parietal lobule contributions to visual word recognition. J Cogn Neurosci. (2015) 27:593–604. 10.1162/jocn_a_00721 25244114

[47] Egner T Monti JM Trittschuh EH Wieneke CA Hirsch J Mesulam MM. Neural integration of top–down spatial and feature–based information in visual search. J Neurosci. (2008) 28:6141–6151. 10.1523/JNEUROSCI.1262-08.2008 PMC6670545 18550756

[48] Liu Y Li L Li B Feng N Li L Zhang X. Decreased triple network connectivity in patients with recent onset post–traumatic stress disorder after a single prolonged trauma exposure. Sci Rep. (2017) 7:12625. 10.1038/s41598-017-12964-6 28974724 PMC5626705

[49] Cavanna AE Trimble MR. The precuneus: a review of its functional anatomy and behavioral correlates. Brain. (2006) 129:564–83. 10.1093/brain/awl004 16399806

[50] Nagahama Y Okada T Katsumi Y Hayashi T Yamauchi H Sawamoto N. Transient neural activity in the medial superior frontal gyrus and precuneus time locked with attention shift between object features. Neuroimage. (1999) 10:193–9. 10.1006/nimg.1999.0451 10417251

[51] Lundstrom BN Ingvar M Petersson KM. The role of precuneus and left inferior frontal cortex during source memory episodic retrieval. Neuroimage. (2005) 27:824–34. 10.1016/j.neuroimage.2005.05.008 15982902

[52] Frings L Wagner K Quiske A Schwarzwald R Spreer J Halsband U. Precuneus is involved in allocentric spatial location encoding and recognition. Exp Brain Res. (2006) 173:661–72. 10.1007/s00221-006-0408-8 16525800

[53] Li HL Chou XM Liang Y Pan T Zhou Q Pei CG. Use of rsfMRI–fALFF for the detection of changes in brain activity in patients with normal–tension glaucoma. Acta Radiol. (2021) 62:414–22. 10.1177/0284185120926901 32571098

[54] Wang Y Shao Y Shi WQ Jiang L Wang XY Zhu PW. The predictive potential of altered spontaneous brain activity patterns in diabetic retinopathy and nephropathy. EPMA J. (2019) 10:249–59. 10.1007/s13167-019-00171-4 31462942 PMC6695464

[55] Tan G Huang X Ye L Wu AH He LX Zhong YL. Altered spontaneous brain activity patterns in patients with unilateral acute open globe injury using amplitude of low–frequency fluctuation: a functional magnetic resonance imaging study. Neuropsychiatr Dis Treat. (2016) 12:2015–20. 10.2147/NDT.S110539 27570455 PMC4986910

[56] Wang CW Chan CL Jin HY. Psychometric properties of the Chinese version of the 25–item national eye institute visual function questionnaire. Optom Vis Sci. (2008) 85:1091–9. 10.1097/OPX.0b013e31818b9f23 18981924

[57] Buffenn AN. The impact of strabismus on psychosocial heath and quality of life: A systematic review. Surv Ophthalmol. (2021) 66:1051–64. 10.1016/j.survophthal.2021.03.005 33773997

